# Back to feedback: aberrant sensorimotor control in music performance under pressure

**DOI:** 10.1038/s42003-021-02879-4

**Published:** 2021-12-16

**Authors:** Shinichi Furuya, Reiko Ishimaru, Takanori Oku, Noriko Nagata

**Affiliations:** 1 Sony Computer Science Laboratories Inc. (Sony CSL), Tokyo, Japan; 2 Sophia University, Tokyo, Japan; 3 NeuroPiano Institute, Kyoto, Japan; 4 School of Science and Technology, Kwansei Gakuin University, Sanda, Japan

**Keywords:** Human behaviour, Motor cortex

## Abstract

Precisely timed production of dexterous actions is often destabilized in anxiogenic situations. Previous studies demonstrated that cognitive functions such as attention and working memory as well as autonomic nervous functions are susceptible to psychological stress in skillful performance while playing sports or musical instruments. However, it is not known whether the degradation of sensorimotor functions underlies such a compromise of skillful performance due to psychophysiological distress. Here, we addressed this issue through a set of behavioral experiments. After artificially delaying the timing of tone production while playing the piano, the local tempo was abnormally disrupted only under pressure. The results suggest that psychological stress degraded the temporal stability of movement control due to an abnormal increase in feedback gain. A learning experiment further demonstrated that the temporal instability of auditory-motor control under pressure was alleviated after practicing piano while ignoring delayed auditory feedback but not after practicing while compensating for the delayed feedback. Together, these findings suggest an abnormal transition from feedforward to feedback control in expert piano performance with psychological stress, which can be mitigated through specialized sensorimotor training that involves piano practice while volitionally ignoring the artificially delayed provision of auditory feedback.

## Abstract (web summary)

In order to establish if the degradation of sensorimotor functions underlies the stress-associated disruption of skilful performance, Furuya et al examined participants undergoing a piano playing task under stress. Their data suggests the occurrence of a stress-induced transition from feedforward to feedback control, which can be mitigated through sensorimotor training involving piano practice while volitionally ignoring the artificially delayed provision of auditory feedback.

## Introduction

Skillful behaviors are readily spoiled in anxiogenic situations such as the Olympic games and music competitions. Suboptimal performance of skillful actions due to psychological stress under pressure is a common problem across trained individuals, such as athletes, musicians, and surgeons, and sometimes affects the future career prospects of these individuals. Psychological stress typically compromises spatiotemporal control of dexterous movements^1–6^. For example, stuttering in speech is exaggerated by psychological stress^7^, which indicates the detrimental effects of the stress on precisely timed execution of motor sequences. Similarly, psychological stress triggers rhythmic distortion of sequential finger movements in musical performance^8–10^. These deleterious effects of psychological stress on motor precision have been extensively argued in terms of their relation to abnormal attention in anxiogenic situations^1,5,11–14^. For example, skilled baseball players were more aware of the direction of bat motion in a more psychologically stressful condition, and it was speculated that the exaggerated attention to the well-learned processes of skill execution led to abnormal modulation of skill execution and thereby increased the movement variability in a stressful condition^5^. While previous studies have reported that cognitive malfunctions such as abnormal attention mediated deleterious effects of psychological stress on skillful behaviors, there remain unclear firstly whether sensory-motor malfunctions underlie the degradation of precision of skillful actions due to psychological stress, and secondly what intervention restores it. Understanding this is essential to shed light on neuropsychological mechanisms subserving the robustness and flexibility of sensorimotor skills in various environments.

A key element of skillful behaviors is the robustness of skills against external perturbation and under uncertainty. The balance between the robustness and flexibility of sensorimotor skills dynamically shifts over a course of learning^15–18^. At the early stage of skill acquisition, a large gain of sensory feedback enables to rapidly correct erroneous motor actions and enhance fast skill improvements with training. Through extensive training, such a skill reaches its plateau and is stabilized^19,20^. The stabilized skill is minimally susceptible to sensory perturbations due to feedforward control, which has been demonstrated in trained individuals^21^. For example, singers but not nonmusicians can keep singing accurately even by artificially shifting the pitch of the voice^22^ or by blocking somatosensory afferent information via vocal-fold anesthesia^23^. Similarly, during piano playing, delayed tone production perturbs the subsequent keystroke motions to a lesser extent in expert pianists than in nonmusicians^24^. Accordingly, it is possible that psychological stress destabilizes experts’ sensorimotor control through aberrantly integrating sensory feedback into control of skillful motor actions.

Here, we addressed the effects of psychological stress on sensorimotor control of expert pianists. We focused on musicians because many musicians suffer from suboptimal musical performance due to psychological stress when performing on the stage^25,26^, prevalent rates of which ranged from 15%^25^ up to 90% of musicians^26^. Indeed, such a problem impairs various aspects of skillful musical performance; for example, our previous research demonstrated abnormal elevation of tempo error while expert pianists were playing the piano in a psychologically stressful situation^9^. In the present study, the stability of sensorimotor control was assessed by the well-established experimental paradigm using the altered sensory feedback, which provided transient sensory perturbation during piano playing^24,27^. By using sensory perturbation, we also tested whether sensorimotor training could alleviate the detrimental effects of psychological stress on the sensory-motor control in the skillful piano performance.

## Results

### Effects of psychological stress on the piano performance

A preliminary experiment with 12 pianists was performed in order to evaluate effects of psychological stress on fine motor control and heart rate on the piano performance. Here, the task was the same as one used for the sensorimotor test without artificially delaying the timing of auditory feedback. The timing error was defined as the absolute error between the predetermined inter-onset interval between two successive tones and observed inter-keystroke interval between two successive strikes, which was then averaged across the strikes. The mean absolute timing error across the participants was 23.5 ± 16.1 ms and 11.3 ± 10.9 ms in the HP and LP condition, respectively. A paired *t*-test confirmed a significant difference between the conditions (*t*(11) = −2.57, *p* = 0.026). The mean heart rate was 79.6 ± 10.0 and 92.0 ± 12.2 beats per minute in the HP and LP condition, respectively. A paired *t*-test yielded a significant difference between the conditions (*t*(11) = −6.56, *p* = 4.08 × 10^−5^). These findings confirm larger timing error of the keystrokes when playing the piano in a more stressful condition.

### Effects of psychological stress on sensorimotor control

**Fig. 1:**
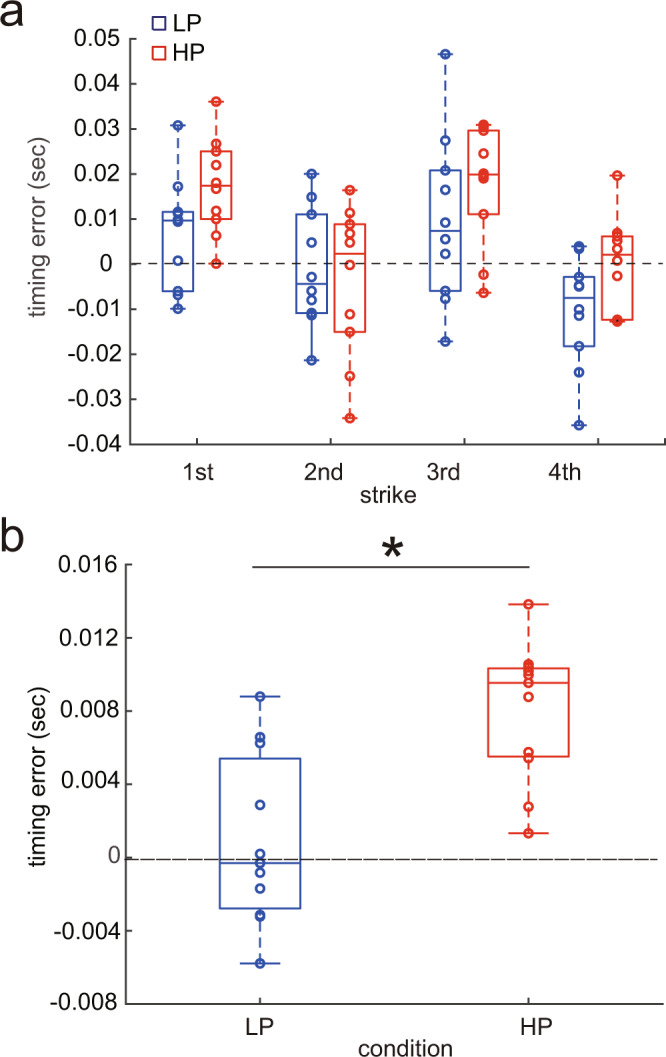
Results of the sensorimotor test of the experiment 1. **a** The timing error of the keypresses over 4 strikes following the provision of transiently delayed tone production, and **b** their mean across these control four strikes in the low-pressure (LP) and high-pressure (HP) conditions. *p < 0.05. The blue and red boxplots indicate the LP and HP conditions, respectively.

Figure 1a illustrates the timing error of the piano keypresses following the provision of the artificially delayed tone production in the pianists during the HP and LP conditions (i.e., the sensorimotor test). A positive value indicates transient slow-down of the local tempo following the perturbation. A two-way repeated-measure ANOVA yielded significant main effects of both condition (F(1, 10) = 19.46, *p* = 0.01, *η*^2^ = 0.073) and strike (F(3, 30) = 6.01, *p* = 0.002, *η*^2^ = 0.27) but no interaction effect of condition and strike (F(3, 30) = 2.11, *p* = 0.120, *η*^2^ = 0.06). To describe the main effect of the condition, the average value across the 4 strikes was computed for each of the HP and LP conditions (Fig. 1b). The effect of psychological stress on the timing error of the keypresses was characterized by the larger timing error in the HP condition compared with the LP condition. These results indicate that pianists slowed down the local tempo of piano playing following the transiently delayed tone production to a larger extent in a more stressful condition. There was no significant correlation of the amount of the stress-induced increase (i.e., a difference in value between the HP and LP conditions) between the heart rate and timing error of the keystrokes by the perturbed auditory feedback (*r* = 0.2742, *p* = 0.4144).

### Effects of psychological stress on motor and perceptual functions

**Fig. 2:**
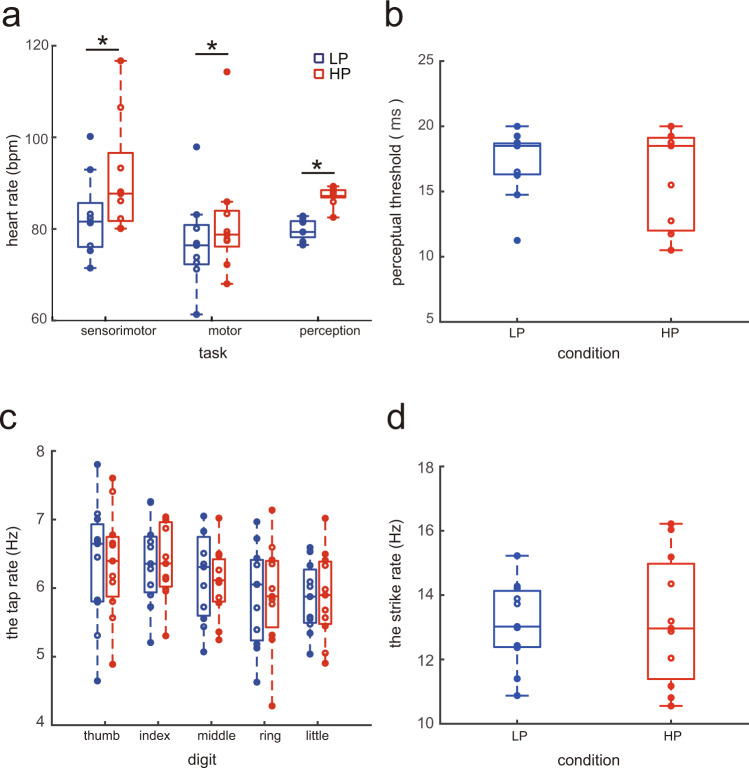
Results of the motor and perception tests and the heart rate at all tests in the experiment 1. **a** Group means of the average heart rate during the sensorimotor control, motor, and perception tests; **b** the perceptual threshold of time duration between two tones; **c** the tap rate during the fastest tapping by each of five digits (i.e., taps per sec); **d** the strike rate by the fingers while playing the short melody excerpt as fast as possible without auditory feedback being provided (i.e., strikes per sec) in the low-pressure (LP) and high-pressure (HP) conditions. *p < 0.05. The blue and red boxplots indicate the LP and HP conditions, respectively.

Figure 2a displays the group means of the average heart rate during each of the sensorimotor control, motor, and perception tasks in the pianists in the HP and LP conditions. During each of the three tasks, the heart rate was higher for the HP condition than for the LP condition. A two-way repeated-measures ANOVA demonstrated a main effect of condition (2 levels: HP and LP) on heart rate (F(1, 7) = 22.71, *p* = 0.002, *η*^2^ = 0.116), but neither a main effect of task (3 levels: task 1–3; F(2,14) = 2.66, *p* = 0.11, *η*^2^ = 0.121) nor an interaction effect (task × condition: F(2, 14) = 2.47, *p* = 0.12, *η*^2^ = 0.008) was evident. A lack of the interaction effect indicates that the pressure effect on the heart rate did not differ between the three different tasks.

Figure 2b illustrates the results of the psychophysics experiment that evaluated the threshold of the auditory perception of the duration of the time interval between two successive tones in pianists in the HP and LP conditions (i.e., the perception test). A paired *t*-test yielded no significant difference between the conditions (*t*(10) = 1.163, *p* = 0.136). This indicates no effects of psychological stress on the auditory perception of the time interval between the two tones.

Figure 2c displays the group means of the maximum tapping rate (i.e., a reciprocal of the mean inter-tap interval) by each of the five digits in pianists during the HP and LP conditions (i.e., the motor test). Here, the tap rate was computed as the reciprocal of the mean inter-keystroke interval between the successive keypresses within a trial. A two-way repeated-measure ANOVA yielded neither an interaction effect between finger and condition (F(4, 40) = 0.154, *p* = 0.96, *η*^2^ = 9.60 × 10^−5^) nor a main effect of condition (F(1, 10) = 3.16 × 10^−4^, *p* = 0.99, *η*^2^ = 5.57 × 10^−7^) but a significant main effect of finger (F(4, 40) = 6.27, *p* = 5.14 × 10^−4^, *η*^2^ = 0.10). This indicates that finger tapping ability did not differ irrespective of the pressure level. Similarly, with respect to the variability of the tap rate within a trial during the fastest finger tapping, neither an interaction effect between finger and condition (F(4, 40) = 0.74, *p* = 0.570, *η*^2^ = 0.015) nor a condition effect (F(1, 10) = 0.40, *p* = 0.539, *η*^2^ = 0.002) were observed. This indicates no effect of psychological stress on the timing precision of the fastest finger tapping movements.

Figure 2d displays the maximum keystroke rate (i.e., a reciprocal of the mean inter-keystroke interval) while playing an excerpt of a melody that consists of 8 successive tones as fast as possible without auditory feedback being provided (i.e., the motor test). A paired *t*-test yielded no significant difference between the conditions (*t*(10) = 0.05, *p* = 0.962). Similarly, with respect to the variability of the keystroke rate within a trial while playing this melody as fast as possible, a paired *t*-test identified no significant difference between the conditions (*t*(10) = −0.37, *p* = 0.721). These results indicate no effects of psychological stress on both the speed and the accuracy of playing a short melody.

**Fig. 3:**
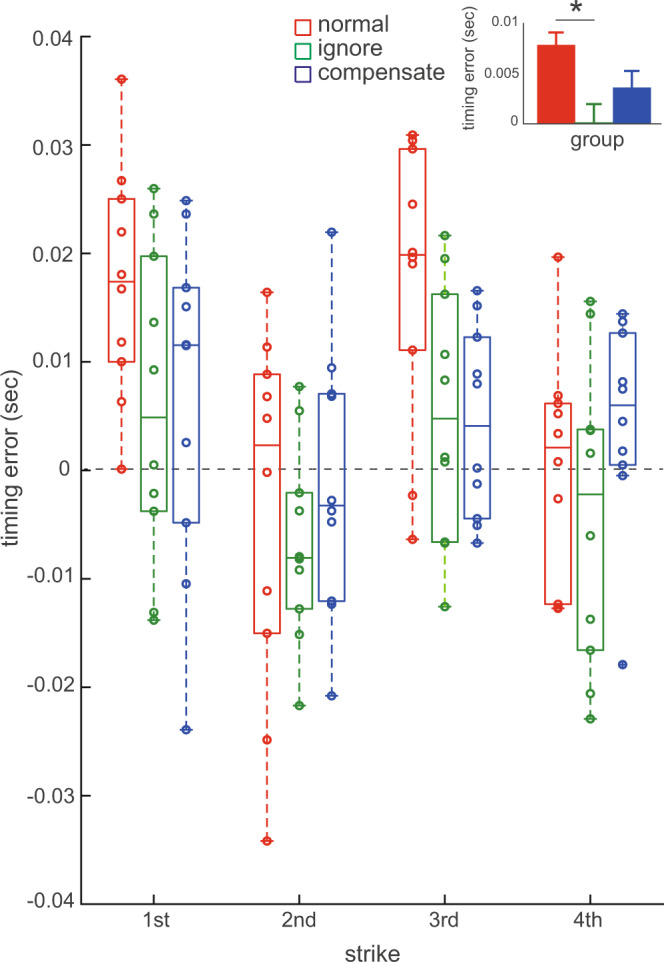
Results of the experiment 2. Group means of differences in the timing error of the four successive keypresses following the provision of transiently delayed tone production between the high-pressure (HP) condition and low-pressure condition before running the intervention (LP_pre_) for the normal (red), ignore (green), and compensate (blue) groups. *x*-axis: four successive strikes immediately after the transiently delayed tone production. The inset indicates the group means of the timing error across the four strikes for each of the normal, ignore, and compensate groups, which was depicted based on a significant group effect but not an interaction effect between group and strike (an error bar indicates one standard error). *p < 0.05. The value 0 indicates no difference in the keypress timing error between the HP and LP_pre_ conditions.

All datapoints used to plot the Figs. 1–3 are included in the Supplementary Data 1.

### Effects of sensorimotor training

Experiment 2 tested whether practicing with the provision of delayed auditory feedback would influence the rhythmic distortion of the keypresses following the perturbation in piano performance under pressure. Figure 3 illustrates differences in the effects of psychological stress on motor responses to transiently delayed tone production among the 3 groups with different interventions (i.e., the normal, ignore, and compensate groups). Here, the value at each strike indicates a difference in the timing error of the keystrokes between the HP condition and the LP_pre_ condition, which was computed to minimize the confounding effects of possible individual differences in the inter-keystroke interval across the groups. A two-way mixed-design ANOVA demonstrated main effects of both group (F(2, 27) = 5.85, *p* = 0.008, *η*^2^ = 0.07) and strike (F(3, 81) = 7.88, *p* = 1.12 × 10^−4^, *η*^2^ = 0.20) but no interaction effect between them (F(6, 81) = 1.17, *p* = 0.33, *η*^2^ = 0.07). A groupwise comparison with corrections for multiple comparisons identified a significant group difference only between the ignore and normal groups (inset of Fig. 3). Furthermore, there was no significant group effect on the timing error of the keystrokes in either the LP_pre_ condition (F(2, 27) = 1.26, *p* = 0.30, *η*^2^ = 0.02) or the LP_post_ condition (F(2, 27) = 2.90, *p* = 0.07, *η*^2^ = 0.05). Together, these findings indicate that the slow-down of the local tempo following delayed tone production under pressure was reduced after short-term piano practice while ignoring the delayed tone production.

To compare the heart rate between the groups, a two-way mixed-design ANOVA was performed by using the group (3 groups) and conditions (LP_pre_ and HP) as independent variables. Neither the interaction (F(2, 30) = 0.01, *p* = 0.986, *η*^2^ = 0.001) nor a group effect (F(2, 30) = 0.19, *p* = 0.829, *η*^2^ = 0.01) was significant, which indicated that the training with the delayed tone production did not affect the pressure effect on the heart rate. In addition, although the experiment 2 did not randomize the order of the LP and HP conditions because of the training experiment paradigm, the heart rate in the HP condition did not differ between the experiment 1 (the sensorimotor test) and experiment 2 (the normal group) (*p* = 0.097), which did not provide physiological evidence supporting for an order effect of the pressure effect.

## Discussion

In the present study, we first tested whether psychological stress yields sensorimotor malfunctions in pianists. The results demonstrated larger rhythmic disruption of the sequential finger movements following transient perturbation of auditory feedback at the more stressful condition, which provided evidence supporting the abnormality in sensory feedback control of movements due to psychological stress. In addition, our experiments did not provide evidence supporting effects of the induced psychological stress on auditory perception of time interval and finger motor precision. These findings further indicate that an increase in the timing error of sequential movements by psychological stress is associated with an abnormal elevation in the sensitivity of motor actions to perceived error (i.e., an elevated feedback gain in sensorimotor control). In a nonstressful musical performance, the feedback gain of sensorimotor control is usually low for pianists but not for musically untrained individuals, as exemplified by a transient increase in rhythmic error of movements following a transient delay in the timing of auditory feedback during piano playing only in the latter individuals^24,27^. Therefore, these findings suggest the de-expertise of sensorimotor control of skilled pianists due to psychological stress, which fits with the idea of choking, describing the failure of skill execution under pressure^28^. In the subsequent experiment, we also tested whether sensorimotor training normalizes the instability of sensorimotor control under pressure. The results of the intervention experiment demonstrated that practicing the piano while ignoring artificially delayed tone production resulted in the amelioration of the unstable sensorimotor control by the stress. Intriguingly, this intervention effect was not observed when playing without psychological stress, which indicates specific training effects on sensorimotor control during playing under stress. Furthermore, such an alleviation of the stress effect was not observed after practicing with compensation for delayed tone production and after practicing without the provision of delayed auditory feedback. Taken together, our results indicated that psychological stress abnormally augmented reliance on sensory feedback in the fine control of sequential finger movements during piano playing, which can be restored specifically by ignoring auditory perturbation during practicing the piano.

A question is why the pianists transiently slowed down the tempo following the perturbed auditory feedback only when playing with psychological stress. One may consider that the feedback gain was elevated due to an increased uncertainty of the perception under pressure. However, the results of our perceptual experiment at least do not support this idea due to a lack of changes in auditory perception of the time interval under pressure. Another possibility is that the pianists became sensitive to the exogenous disturbance of auditory feedback in piano playing only under pressure, which had the pianists fail to ignore the disturbance. However, this is also not likely because of no difference in the amount of elevation in the heart rate due to the pressure between the present experiment with auditory perturbation (7.2 ± 2.2 bpm) and our previous experiment that used the same task without auditory perturbation (10.8 ± 6.8 bpm)^9^ (*p* = 0.129). One plausible explanation is that the transient slow-down of the local tempo following the perturbation reflected attempts to lower accuracy demands on the performance by leveraging a tradeoff between the speed and the accuracy of movements^29^. This idea is compatible with elevated coactivation between the finger flexor and extensor muscles under pressure, which is considered a coping strategy for accuracy demand^8,10^. A recent study also proposed a role of muscular coactivation in feedback control of movements^30^. The shift from feedforward to feedback control in the anxiogenic situation can affect reduced movement individuation between the fingers under pressure^9^, because the elevation of the muscular coactivation increases the stiffness of the finger muscles connected with multiple fingers and thereby increases biomechanical constraints on the individuated finger movements^31^. Importantly, the reduced movement individuation between the fingers was associated with an increase in the timing error of the piano performance under pressure^9^, which suggests that elevating feedback gain in the stressful condition played no beneficial role in accurate musical performance. In addition, the transient slow-down of the local tempo after delayed tone production under pressure was also unlikely to contribute to accurate tempo control because the maintenance of the global tempo after delayed tone production requires a transient speed-up of the local tempo to compensate for the transient delay^27^. Taken together, the increased reliance on feedback control under pressure may be not the error correction in the uncertain situation but a mere response to an increased stress.

Our observation indicating the increased gain of sensorimotor feedback control of movements under pressure fits well with the explicit monitoring theory^1,3^. The theory proposes that after an appraisal of performance pressure, attention shifts exaggeratedly toward the procedural process of motor performance, which eventually collapses skillful performance. Previous studies reported that this attentional shift to the automatized skill execution process is detrimental, particularly for skilled individuals who can perform the well-learned procedural skill without explicitly attending to its underlying process^5,11^. Although this theory explains well that skilled players are better at being aware of a course of skill execution in the higher stressful situation^5^, the present study provides another but not exclusive viewpoint that a shift in sensorimotor control from feedforward to feedback control mediates the deleterious effect of pressure on performance. This idea is compatible with a recent finding of a pressure-induced increase in the activation of the anterior cingulate cortex that is responsible for monitoring motor performance^4^.

One may speculate that the present observation can be also explained by a change in the Kalman gain under pressure in the optimal feedback control^32,33^. In this theory, the Kalman gain in sensorimotor control is modulated based on uncertainty of the sensory feedback (i.e., state estimation). Pianists can accurately predict the sensory consequences of the movements by means of the forward model^34^. It is possible that the pressure causes an erroneous state estimation and thereby increases in the Kalman gain, which needs to be investigated in a further experiment that manipulates the uncertainty of the feedback.

Another possible interpretation is that psychological stress augmented responses to deviation from prior expectation of timing of tone production during playing the piano^35^, which can be formed through years of extensive piano training. Exposure to the delayed auditory feedback during the sensorimotor training might then shift the prior so that the pianists take into consideration temporal deviation of tone production to a larger extent, and thereby attenuate movement disruption elicited by the auditory perturbation. This interpretation is also compatible with a lack of changes in the auditory perception due to the stress, because it is unlikely that pianists form some specialized prior for the perceptual task that is unfamiliar to them.

Our intervention study clearly demonstrated that practicing the piano while ignoring delayed tone production resulted in robust tempo control against artificially delayed tone production in piano playing under pressure. Such a robust sensorimotor control characterizes the piano performance without explicit psychological stress^24^. Therefore, the abnormal elevation of the sensorimotor feedback gain under pressure can be normalized through this way of practicing. The lack of a significant difference in the tempo error between the training group that compensated for the delayed tone production and the control group further indicates a specific effect of practicing while ignoring the delayed tone production on the performance instability under pressure. This implicates the potential of the practice of ignoring the auditory perturbation as a training method for stabilizing tempo control in piano performance under pressure. Previous studies demonstrated that skilled singers are better at ignoring the auditory perturbation in singing than nonmusicians^22^, similar to pianists^24^. The present sensorimotor training may enable pianists to maintain low feedback gain in an uncertain and stressful situation.

There are several possible limitations of the present study. First, because a repetition of playing under pressure potentially causes habituation to psychological pressure, we did not additionally evaluate piano performance without the provision of delayed tone production in the stressful condition following the intervention experiment. However, this limits the understanding of a direct relationship of the loss of robustness in sensorimotor control with temporal inaccuracy of piano performance in stressful situations, which needs to be further explored. Second, although we evaluated the time interval perception at rest, we did not evaluate time perception while playing the piano, which might be affected by psychological stress, unlike the time perception at rest. Third, it was ideally necessary to balance the task difficulty between the perception, motor, and sensorimotor tests, in order to compare the effect of psychological pressure on performance of these tasks, which was however difficult to realize. Fourth, there was a discrepancy in terms of effects of the stress induction paradigm between our previous and present studies^8,9^. These include whether the experimenter who evaluated the performance was a pianist (not in our first study^8^), whether the experimenter pretended to write on a blank sheet during the evaluation (only in this study), and/or whether the participants were all Japanese (in our second^9^ and the present studies) or not^8^. Fifth, as a physiological measure of the induced stress, the present study used the average heart rate based on our previous observation^9^. However, several studies also reported sensitivity of the other cardiac measures such as heart rate variability to psychological stress in musicians^36^, which should be assessed in future studies. Finally, we did not examine changes in finger muscular co-contraction under pressure through ignoring practice, which should be assessed in a future study.

## Methods

### Participants

In the present study, 11 (7 females, 25.4 ± 4.9 yrs old) and 30 (23 females, 24.1 ± 6.0 yrs old) right-handed expert pianists with no history of neurological disorders participated in experiments 1 and 2, respectively. In addition, before running these experiments, a preliminary experiment was performed with another 12 pianists (9 females, 21.9 ± 8.7 yrs old) to confirm effects of the psychological stress on fine motor control and autonomic nervous functions in the piano performance. All of the pianists majored in piano performance in a musical conservatory and/or had extensive and continuous private piano training under the supervision of a professional pianist/piano professor. Inclusion criteria were that the age at which the participants had started to play the piano was before 8 years old, and that the total amount of the piano practice until the age twenty was more than 10,000 h, which confirmed their experience of deliberate piano practice. In accordance with the Declaration of Helsinki, the experimental procedures were explained to all participants. Informed consent was obtained from all participants prior to participating in the experiment, and the experimental protocol was approved by the ethics committee of Sophia University.

### Experimental design

The present study consists of 2 experiments under both low-pressure (LP) and high-pressure (HP) conditions. To elicit psychological stress, at the HP condition, another experimenter stood next to the participant, and monitored and evaluated the performance. Before initiating the HP session, the experimenter told the participants that he/she would evaluate the performance. During each test, the experimenter pretended to write on a blank sheet so that the participant could consider having the performance being evaluated. The performance was also videotaped by a camera put in front of the performer. To circumvent confounding effects of familiarization with the experimental situation and stress induction, participants were different across the experiments.

Experiment 1 was primarily designed to test whether psychological stress influences sensory feedback control of movements in piano performance. In addition, the experiment 1 assessed the stress effects on the agility and accuracy of movements and perception of the duration of time interval based on auditory stimuli. In total, 3 tests were carried out in the experiment 1, which assessed the auditory feedback control of movements (“*sensorimotor test*”), finger motor agility and accuracy (“*motor test*”), and perceptual threshold for discrimination of the auditory time interval (“*perception test*”) in the LP and HP conditions (i.e., an within-subject design with a factor of “psychological stress”). We investigated not only the sensorimotor control but also motor functions and temporal perception under pressure, because the latter two can also malfunction due to psychophysiological distress. For example, induced psychological stress elevated finger force fluctuation during a precision grip^37^. In addition, fear and anxiety distort time perception, such as interval timing and simultaneity^38–40^. However, most of these studies targeted individuals without any history of extensive training, which questions the motor functions and time perception of experts under pressure. We assessed the agility and accuracy of movements and perception of the duration of time interval based on auditory stimuli, because it is impossible to assess the whole spectrum of perceptual and motor functions. In addition, we recognize the limitation that it is hard to experimentally induce psychological stress at the perceptual and motor tests in the perfectly same way as playing the piano.

The sensorimotor test asked the participants to play a short melody extracted from Op. 10 No. 8, composed by Frédéric François Chopin, with only the right hand (the first 14 bars) on an acoustic piano with MIDI sensors (YUS1SH, Yamaha, Co.) at a tempo of 116 bpm (inter-keystroke interval = 129.3 msec) and at the loudness of *mezzo-forte*. This piece was chosen first because it is technically challenging enough to elicit anxiety for many pianists (note that this piece has been played at many piano competitions and during the entrance exams for many musical conservatories) and second because it requires high timing precision of the keystrokes during fast tempo performance with little effects of emotional or aesthetic expression^9^. In addition, we previously confirmed disruption of performance of the present task by the induced psychological stress^9^. The target melody was to be played with legato touch, meaning that a key was not released until the next key was depressed. On the day of the experiment, participants were initially asked to become familiar with the piano by practicing for 10 min, without using a metronome, prior to the experiment. After the familiarization session, all participants were able to play the target melody with the predetermined fingering at the target tempo without pitch errors. During the sensorimotor test, prior to the task performance, 16 metronome tones were provided to a cue of the tempo (i.e., 116 bpm), and then the pianists were asked to play without the metronome sounds. While playing the target melody, the timing of tone production was artificially delayed by 80 ms over 4 successive strikes (i.e., delayed auditory feedback) as auditory perturbation^24,27,41^, which occurred 3 times within the melody. The notes with delayed tone production were not told to the pianists in advance and were randomized across the pianists so that each pianist could not predict which keystrokes elicited delayed tone production. It is obvious that such an artificially delayed tone production does not occur in a real piano performance on stage, but this empirical technique provides a unique opportunity of evaluating stability of the movements through assessing the relationship between the input (i.e., perturbation) and output (i.e., reaction)^27^.

The motor test asked the participants first to perform repetitive strikes of a piano key with each of five digits of the right hand as fast and accurately as possible for 5 s (“the fastest single finger tapping”)^42^ and second to play the first 8 tones of the piece of Op. 10 No. 8, composed by Frédéric François Chopin (i.e., the same piece used for the sensorimotor test), on the piano as fast and accurately as possible (“the fastest piano performance”). Here, the second motor task consisted of tones of A5, G5, F5, C5, A4, G4, F4, and C4, which were played with the fingering of the ring, middle, index, thumb, ring, middle, index, and thumb, respectively. During the performance of these motor tests, the piano tones were deprived (i.e., muted) so that auditory feedback could not affect movement execution. These motor tests evaluated both the speed and the timing precision of the sequential keystrokes with a single finger and with multiple fingers by computing the mean and the standard deviation of the inter-keystroke interval across the strikes within a trial. Each task was repeated twice.

The perception test provided 2 sets of auditory stimuli, each of which consisted of 2 successive tones. The first “reference” stimulus was 2 successive tones with the inter-tone duration of 129.3 msec (i.e., a tempo of 116 beats per minute (bpm), which is same as one in the sensorimotor task), whereas the second stimulus was 2 successive tones with the inter-tone duration specified according to the staircase method that we used in our previous study^43^. After providing the 2 stimuli, participants were asked to answer whether the inter-tone duration of the second stimulus was shorter than that of the first stimulus. Here, the inter-tone duration of the second stimulus was determined by a three-down one-up staircase method. At the first trial, the inter-tone duration was set to 123.0 ms. At the next trial, the inter-tone duration was decreased or increased by 2 ms, according to whether correct answers were repeated 3 times or an answer was incorrect once, respectively. The test was terminated when 4 peaks and 4 troughs were measured, and the average of the last 2 peaks and 2 troughs of the inter-tone duration was defined as the perceptual threshold of the auditory time-interval discrimination. In the present perceptual test, we employed this method rather than estimating the psychometric function because the former was quicker in the threshold detection, which we considered suitable for the present study that concerns a decrease of the pressure effect over time.

For experiment 1, the order of the sensorimotor, motor, and perception tests was randomized across the participants. In the LP condition, the experimenter sat behind each pianist and out of sight. In the HP session, the stress induction was performed in a manner used in our previous study^8^. Another experimenter appeared and adopted a strict and unfriendly behavior; this experimenter stood next to the participant and monitored and evaluated the performance. During each test, the experimenter pretended to write on a blank sheet so that the participant could consider having the performance being evaluated. In addition, the performance was videotaped by a camera mounted in front of the performer. Prior to initiating the HP session, the experimenter told the participants that he/she would evaluate the performance. This experimental design was validated and adopted in our previous studies^8,9^. In addition, in the present study, the participants were asked in each of the LP and HP conditions about to what extent they perceived the stress by using the 7-point Likert scale (a larger value indicates higher stress). The results of the subjective rating were 2.0 ± 1.0 and 5.7 ± 0.8 points in the LP and HP conditions, respectively (*p* = 2.06 × 10^−6^), which confirmed a significant difference in the stress level between the present experimental conditions. The order between the LP and HP conditions was randomized across the participants, because the stress manipulation was a within-participant factor in the present study. Between the conditions, the participants took rest for about ten minutes until the heart rate returned to the baseline level (see details on heart rate assessment/analysis below in the Data Acquisition section).

Experiment 2 was designed to assess the effects of short-term piano practicing with the provision of transiently delayed auditory feedback on the disruption of piano performance following auditory perturbation. The experiment consisted of 4 successive sessions in the following order: playing in the LP condition (LP_pre_), intervention, playing in the LP condition (LP_post_), and playing in the HP condition (HP). Thirty pianists were randomly assigned to 3 groups undergoing different interventions. At the intervention session, they played the first 8 notes of the target melody over 200 trials. The first control group was asked to play the 8 notes without any artificial delay in the timing of the provision of auditory feedback (“normal” group). The second group was instructed to play while ignoring the 80-ms delayed tone production, which occurred at each of the last 4 notes (“ignore” group). The third group was instructed to play while compensating for the delayed tone production at each of the last 4 notes so that the tones could sound with consistent inter-tone intervals across the 8 notes (“compensate” group). This means that the pianists in this group had to strike the keys 80 ms prior to the timing at which the tone should be elicited at the latter 4 strikes. Prior to each trial, 4 metronome tones were provided as a cue for the target tempo, and then the participants began playing without the metronome cue. The intervention and its instruction was designed based on previous studies in which participants were instructed to either ignore or compensate for the altered auditory feedback during movements^22,44–46^.

### Data acquisition

We recorded MIDI data from the piano by using a custom-made script written in JAVA^24^. This script allows for artificially delaying the timing of tone production during piano playing by waiting for a predetermined duration until the MIDI signal is elicited to the piano. Based on the MIDI data collected by this script during the experiments, we derived the time at which each key was depressed and released. Based on this information, the inter-keystroke interval was computed (note that this is a difference in timing between two successive keypresses, but not a difference in tone onset between two successive tones). The electrocardiogram data of each pianist was also recorded during the task performance in the experiments (Mio Slice, Mio Global Inc.) and data were analyzed in an offline manner. The electrocardiogram data were then expressed in bpm (beat per minute) for each R–R interval. In experiment 1 only, the heart rate recordings from 3 pianists were contaminated by unpredictable noises, resulting in missing data; therefore, the data from these pianists were not used for the subsequent analyses.

### Data analysis

Using information on the inter-keystroke interval during piano playing, we defined the timing error as the temporal gap of the inter-keystroke intervals between the measured and ideal presses. Here, the inter-keystroke interval of the ideal presses was defined based on the tempo provided by the metronome. The timing error for each of the inter-keystroke intervals was then averaged across the strikes for each participant under each of the HP and LP conditions. For the perception test of experiment 1, the perceptual threshold was computed based on the aforementioned manner (i.e., a staircase method). For the motor test of experiment 1, the inter-keystroke interval was first computed as a difference in the timing of the keypress between two successive keystrokes, and secondly averaged across the keypresses within a trial. The derived mean inter-keystroke interval within a trial was averaged between the two trials, and the reciprocal of the averaged inter-keystroke interval was defined as the tap rate in Hz (i.e., taps per second). Therefore, note that this tap rate does not mean the number of taps to be directly counted. Note that source data for figure are provided with the paper as the Supplementary Data.

### Statistics

For datasets of the perception test and motor test (the fastest piano performance) in experiment 1, a paired *t*-test was performed for a comparison between the HP and LP conditions (alpha level = 0.05). If the dataset did not follow the normal distribution based on the Kolmogorov–Smirnov test, a nonparametric Wilcoxon test was performed. For the heart rate in experiment 1, a two-way repeated-measures ANOVA was performed with task (3 levels: perception, motor, sensorimotor tests) and condition (2 levels: LP and HP) as within-subject independent variables. For the timing error at the sensorimotor test in experiment 1, a two-way repeated-measures ANOVA was performed with strike (4 levels: the first, second, third, and fourth strikes following provision of the delayed tone production) and condition (2 levels: LP and HP) as within-subject independent variables. For the fastest finger tapping at the motor test in experiment 1, a two-way repeated-measures ANOVA was performed with digit (5 levels: thumb, index, middle, ring, and little fingers) and condition (2 levels: LP and HP) as within-subject independent variables. With respect to experiment 2, a two-way mixed-design ANOVA was performed with group (3 levels: normal, ignore, and compensate) and strike (4 levels: the first, second, third, and fourth strikes following the provision of delayed tone production) as independent variables. Mauchly’s test was used to test for sphericity prior to performing each ANOVA, and for nonspherical data, the Greenhouse–Geisser correction was performed. Post hoc tests with correction for multiple comparisons^47^ were performed in the case of significance. These statistical analyses were performed with R statistical software (ver. 3.2.3.).

### Reporting summary

Further information on research design is available in the Nature Research Reporting Summary linked to this article.

## Supplementary information

Description of Additional Supplementary Files

Supplementary Data 1

Reporting Summary

## Data Availability

The data that support the findings of this study are available on request from the corresponding author [S.F.]. The data are not publicly available due to them containing information that could compromise research participant privacy.

## Code availability

Codes will be provided upon request.
