# Supplementary material for: Back to feedback: aberrant sensorimotor control in music performance under pressure
Source: Commun Biol. 2021 Dec 16;4:1367. doi: 10.1038/s42003-021-02879-4 (PMC8677784; doi:10.1038/s42003-021-02879-4)
Supplement: Supplementary file 1 — Description of Additional Supplementary Files [file 42003_2021_2879_MOESM1_ESM.pdf]

**Description of Additional Supplementary Files**

**File name:** Supplementary Data 1

**Description:** Summarizes all datapoints used for plotting the Figures 1-3.
