# Supplementary material for: Back to feedback: aberrant sensorimotor control in music performance under pressure
Source: Commun Biol. 2021 Dec 16;4:1367. doi: 10.1038/s42003-021-02879-4 (PMC8677784; doi:10.1038/s42003-021-02879-4)
Supplement: Supplementary file 3 — Reporting Summary [file 42003_2021_2879_MOESM3_ESM.pdf]

nature portfolio

Corresponding author(s): *Double-blind peer review submissions: write DBPR and your manuscript number here instead of author names.*

Last updated by author(s): *YYYY-MM-DD*

Reporting Summary

Nature Portfolio wishes to improve the reproducibility of the work that we publish. This form provides structure for consistency and transparency in reporting. For further information on Nature Portfolio policies, see our Editorial Policies and the Editorial Policy Checklist.

Statistics

For all statistical analyses, confirm that the following items are present in the figure legend, table legend, main text, or Methods section.

n/a	Confirmed	
☐	☒	The exact sample size (*n*) for each experimental group/condition, given as a discrete number and unit of measurement
☐	☒	A statement on whether measurements were taken from distinct samples or whether the same sample was measured repeatedly
☐	☒	The statistical test(s) used AND whether they are one- or two-sided<br>*Only common tests should be described solely by name; describe more complex techniques in the Methods section.*
☐	☒	A description of all covariates tested
☐	☒	A description of any assumptions or corrections, such as tests of normality and adjustment for multiple comparisons
☐	☐	A full description of the statistical parameters including central tendency (e.g. means) or other basic estimates (e.g. regression coefficient) AND variation (e.g. standard deviation) or associated estimates of uncertainty (e.g. confidence intervals)
☐	☒	For null hypothesis testing, the test statistic (e.g. *F*, *t*, *r*) with confidence intervals, effect sizes, degrees of freedom and *P* value noted<br>*Give P values as exact values whenever suitable.*
☒	☐	For Bayesian analysis, information on the choice of priors and Markov chain Monte Carlo settings
☒	☐	For hierarchical and complex designs, identification of the appropriate level for tests and full reporting of outcomes
☐	☒	Estimates of effect sizes (e.g. Cohen's *d*, Pearson's *r*), indicating how they were calculated

Our web collection on statistics for biologists contains articles on many of the points above.

Software and code

Policy information about availability of computer code

Data collection	MATLAB (Mathworks Inc.)
Data analysis	MATLAB (Mathworks Inc.), R

For manuscripts utilizing custom algorithms or software that are central to the research but not yet described in published literature, software must be made available to editors and reviewers. We strongly encourage code deposition in a community repository (e.g. GitHub). See the Nature Portfolio guidelines for submitting code & software for further information.

Data

Policy information about availability of data

All manuscripts must include a data availability statement. This statement should provide the following information, where applicable:

- Accession codes, unique identifiers, or web links for publicly available datasets
- A description of any restrictions on data availability
- For clinical datasets or third party data, please ensure that the statement adheres to our policy

All data generated or analysed during this study are included in this published article (and its supplementary information file).

Field-specific reporting

Behavioural & social sciences study design

All studies must disclose on these points even when the disclosure is negative.

Study description	quantitative experiment
Research sample	In the present study, 11 (7 females, 25.4 ± 4.9 yrs old) and 30 (23 females, 24.1 ± 6.0 yrs old) right-handed expert pianists with no history of neurological disorders participated in experiments 1 and 2, respectively. We recruited these pianists through an on-line questionnaire, and randomly sampled based on the inclusion criteria.
Sampling strategy	We randomly sampled the participants in the study, and the number of the participants was defined according to the power analysis based on our statistical test used for the study.
Data collection	We used a PC and an electric piano to collect data on piano keystrokes. In addition, we used a mobile heart-rate sensor. An experimenter was blinded on the hypothesis.
Timing	From Oct 1, 2018 until July 31, 2020
Data exclusions	The heart rate recordings from 3 pianists were contaminated by unpredictable noises, resulting in missing data; therefore, the data from these pianists were not used for the subsequent analyses.
Non-participation	zero
Randomization	Participants were randomly allocated to different intervention groups in the experiment 2.

Reporting for specific materials, systems and methods

We require information from authors about some types of materials, experimental systems and methods used in many studies. Here, indicate whether each material, system or method listed is relevant to your study. If you are not sure if a list item applies to your research, read the appropriate section before selecting a response.

Materials & experimental systems

n/a	Involved in the study	
☒	☐	Antibodies
☒	☐	Eukaryotic cell lines
☒	☐	Palaeontology and archaeology
☒	☐	Animals and other organisms
☐	☒	Human research participants
☒	☐	Clinical data
☒	☐	Dual use research of concern

Methods

n/a	Involved in the study	
☒	☐	ChIP-seq
☒	☐	Flow cytometry
☒	☐	MRI-based neuroimaging

Human research participants

Policy information about studies involving human research participants

Population characteristics	See above.
Recruitment	Online questionnaire
Ethics oversight	Sophia University

Note that full information on the approval of the study protocol must also be provided in the manuscript.
